# The effect of complete replacing sodium with potassium, calcium, and magnesium brine on sodium‐free ultrafiltration Feta cheese at the end of the 60‐day ripening period: Physicochemical, proteolysis–lipolysis indices, microbial, colorimetric, and sensory evaluation

**DOI:** 10.1002/fsn3.2050

**Published:** 2020-12-02

**Authors:** Razieh Zonoubi, Mohammad Goli

**Affiliations:** ^1^ Department of Food Science and Technology Isfahan (Khorasgan) Branch Islamic Azad University Isfahan Iran; ^2^ Laser and Biophotonics in Biotechnologies Research Center Isfahan (Khorasgan) Branch Islamic Azad University Isfahan Iran

**Keywords:** Na‐salt substitution, overall acceptability, proteolysis–lipolysis indices, sodium‐free UF‐feta cheese, textural properties, whiteness index

## Abstract

The effect of complete substitution of NaCl with KCl, MgCl_2_, and CaCl_2_ in brine used for the ripening stage in Na‐free ultrafiltration (UF) Feta cheese making investigated. The chemical, microbial, textural, colorimetric, and sensory evaluation did at the end of the 60‐day ripening period. As the ripening period of the cheese increased, the amount of acidity and total solid significantly increased while pH and moisture significantly decreased. All chloride salts exerted a significant antimicrobial effect on the fermentation growth cycle; particularly, CaCl_2_ showed a similar effect to NaCl, while KCl and MgCl_2_ were progressively less inhibitory. The highest hardness and syneresis on the first day seen in the samples containing sodium chloride and the lowest hardness and syneresis on the sixty day recognized in the samples containing magnesium chloride. There was no significant difference in whiteness index for monovalent salts in the first and sixtieth days of storage, and of course, this difference was not significant between divalent salts. There was no significant difference in overall acceptance score between sodium and potassium brine, although these two treatments had a significant difference compared with the others. There was no significant difference in the overall acceptance of cheeses stored in calcium and magnesium brine.

## Graphical abstract

The ripening period significantly increased and decreased the acidity‐total solid and the pH‐moisture, respectively. All chloride salts exerted a significant antimicrobial effect on the fermentation growth cycle. CaCl_2_ had a similar effect to NaCl, while KCl and MgCl_2_ had a less inhibitory effect on the fermentation growth cycle. The highest hardness and syneresis on the first day seen in the samples containing sodium chloride. The lowest hardness and syneresis on the sixty‐day recognized in the samples containing magnesium chloride. There was no significant difference in the whiteness index for monovalent salts in one and 60‐day ripening periods. There was no significant difference in the whiteness index for divalent salts in one and 60‐day ripening periods. There was no significant difference in the overall acceptance of cheeses stored in Na and K brine. There was no significant difference in the overall acceptance of cheeses stored in Ca and Mg brine.

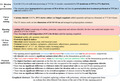

## 1 INTRODUCTION

Cheese, as the main dairy product, has generally consumed in the world due to its high nutritional value. Iranian ultrafiltration (UF) Feta cheese, soft cheese with an acidic and salty taste; minimum of 34% (w/w) total solids; protein content of 12%, maximum acidity of 1.80% lactic acid; and a pH of 4.60 to 5.20, is made in the lactic fermentation process by action of some lactic acid bacteria strains and renin, on the cow milk. This product is high perishable due to growth of both pathogens and spoiling microorganisms on its surface. Therefore, cheese has a low shelf life, and its lower pH and storage in sodium brine help to increase its shelf life up to 1–2 month in the refrigerator temperature (ISIRI, 2015; Nottagh et al., 2020; Omrani Khiabanian et al., 2020). Among the types of cheeses available in the market, UF‐Feta cheese has the highest per capita consumption in Iran due to its high production efficiency and lower price than other cheeses. This type of enzymatic‐coagulated cheese resulted in the concentrated milk, up to approximately 30% of dry matter containing highly nutritious whey proteins, has a soft texture with a uniform structure (Kulozik, 2019).

The sodium required to maintain physiological and biological functions in the body is 0.18 to 0.23 g/day; however, the global mean consumption of sodium in 2010 was approximately 3.95 g/day. Among various dairy products, cheese contains a high amount of dietary sodium, which results from the addition of table salt during the manufacturing of cheese. The sodium content in cheese varies from 40 to 800 mg/100 g for most types of natural cheese, whereas in processed cheese, it can be as high as 1,500 mg/100 g. Moreover, the world health organization (WHO) has established a global target of a 30% reduction in sodium chloride intake by the year 2025 (Bansal & Kumar‐Mishra, 2019; WHO, 2012).

The human hypertension disease results in a multifactorial combination of genetic and environmental agents. There is strong evidence of a causal relationship between high salt intake and high blood pressure and risk of stroke in humans and animals (Frisoli et al., 2012; Pierre et al., 2005; Youssef et al., 2017). The WHO recommends a population salt intake less than 5 g/day‐person. In contrast, increased potassium intake reported to protect stroke, high blood pressure, heart harmony problems, and kidney failure. The additional use of potassium chloride to partially replacing with sodium chloride could help reduce the sodium content (WHO, 2012). Potassium plays a parallel role with sodium, and the balance between the two elements is important in the control of blood pressure. However, the use of potassium chloride is mainly restricted due to its bitter taste (Bansal & Rani, 2014; EUFIC, 2006; Youssef et al., 2017). Magnesium decreases intracellular sodium and calcium, which causes low blood pressure. Magnesium is a natural calcium channel blocker, blocks sodium attachment to vascular smooth muscle cells, increases vasodilating prostaglandin, cooperatively binds potassium, increases nitric oxide, improves endothelial dysfunction, causes vasodilation, and reduces blood pressure (Houston, 2011; Youssef et al., 2017). Animal, observational studies, trials, and meta‐analyses studies confirmed that dietary calcium influences on blood pressure. Meta‐analyses advised that calcium supplementation (400–2,000 mg/day) results in moderate reductions in systolic and diastolic blood pressure. In most humans, healthy blood pressure depends upon a balance of both Na: K and Mg: Ca ratios at both cellular and whole‐body levels (Appel, 2009; Youssef et al., 2017). Many kinds of cheese contain a high concentration of sodium chloride, in which the substitution of sodium chloride by potassium chloride has been applied. Potassium chloride revealed a high potential for used as a salt replacer without changing the chemical properties of cheeses. A blend of 50% sodium chloride and 50% potassium chloride was suggested as a salt replacer in processed cheese (Patel et al., 2013).

This study aimed to produce sodium‐free UF‐Feta cheese for the hypertensive patients by complete substitution of sodium chloride with potassium chloride or other salts such as magnesium and calcium chloride in brine. Consequently, the effect of these minerals on physicochemical, proteolysis–lipolysis indices, microbial, colorimetric, and sensory evaluation at the end of the 60 days ripening period was studied.

## 2 MATERIALS AND METHODS

### 2.1 Materials

*Lactococcus lactis Subsp. lactis* and *Lactococcus lactis Subsp. cremoris* (Delvo®Cheese CT‐type starter culture, DSM’s Delvo®, France) and fungal coagulants from *Rhizomucor miehei* (DSM’s Fromase®, France) and the brine salts including calcium chloride, magnesium chloride, potassium chloride, and sodium chloride (Merck, Germany) were used in the production of ultrafiltration cheese.

### 2.2 Stages of UF‐Feta cheese production

After low‐fat cow milk heat processing at 75°C for 15 s, the milk (1% fat) concentrated by membrane filtration (DSS Company, Denmark) at 50°C to 27% final brix. The retentate phase subjected to a homogenization process at a pressure of 50 to 60 bar, and then, the pasteurization heat treatment was performed at 75°C for 1 min and cooled to 32°C. Then, calcium chloride 0.015%, direct vat set (DVS) starter culture, and fungal coagulants added separately. Then, the mixture was put into closed containers and kept in a fermenter at 37°C for 8 hr to allow coagulation to take place. Then, the cheese curds were cut into dimensions of 10 × 10 × 10 cm and arranged in polypropylene containers. After adding 10% brine (comprising of sodium, potassium, magnesium and calcium chloride), the door was sealed and samples were placed at 25°C for 24 hr. They then kept at cold temperature (5–8°C) for 60 days until the ripening completed. Finally, physicochemical, textural, and colorimetric properties and bacterial counting of ultrafiltration cheese on the first and sixtieth day were assessed.

### 2.3 Physicochemical analysis of UF‐Feta cheese

Cheese samples were analyzed for titratable acidity according to AOAC (2002). The pH of cheese samples was determined using a GP353 ATC pH meter (EDT Instruments) (Ardo & Polychroniadou, 1999).The moisture content and total solid of cheese samples was determined by drying at 102 ± 2°C to a constant weight (Anyfantakis, 1992). The salt concentration of cheese samples was determined according to AOAC (2002). The different nitrogen fractions of cheese samples were determined using the Kjeldahl method (AOAC, 2002). Cheese samples were analyzed for total nitrogen (TN) and water‐soluble nitrogen (WSN). WSN of cheese samples was expressed as a percentage of the TN (g/100 g). Protein content of cheese samples was calculated as 6.38 × (TN ‐ NPN). Nonprotein nitrogen (NPN) is the fraction of TN soluble in 12 g/100 ml trichloroacetic acid. The ripening index (RI) or proteolysis index (PI) also was calculated as a percentage of the ratio of WSN to TN. Lipolysis index (LI) or acid degree value (ADV) also was calculated as mEq. KOH was to neutralize the free acidity of 100 g of cheese oil (Ardo & Polychroniadou, 1999). Analyses were carried out in triplicate.

### 2.4 Texture Profile Analysis of UF‐Feta cheese

Texture profile (hardness, springiness, cohesiveness, adhesiveness, gumminess, and chewiness) was analyzed according to Ayyash et al. (2011) with some modifications. Cylinders of 25 × 20‐mm cheeses were cut from UF‐Feta cheese blocks at the center. Specimens were kept in a refrigerator at 4°C overnight, followed by determination of texture profile. Textural parameters were measured using an Instron universal testing machine (model 5564; Instron Ltd.). The samples were compressed to 50% of their heights using a 100‐N load cell with a flat plunger and the crosshead movement was adjusted to 10 mm/min. Double compression was achieved, and the data were collected using Merline software. Analyses were carried out in quadruplicate.

### 2.5 Color assessment of UF‐Feta cheese

The first, the color parameters, that is, L*, a*, and b* values, were analyzed for UF‐Feta cheese using the Hunter laboratory machine (UltraScanvis, US‐Vis 1,310, USA). The L* measures lightness and varies from zero (black) to 100 (white), a* measures tonality from + a (red) to –a (green), while b* measures tonality from + b (yellow) to –b (blue). The second, whiteness index (WI) and browning index (BI), which represents the purity of white and brown color, respectively, was calculated according to below equations (Farokhian et al., 2016):

$$\Delta E\;=\;\sqrt{{\left(b_{60th}‐b_{1st}\right)}^{2}+{\left(a_{60th}‐a_{1st}\right)}^{2}+(L_{60th}‐L_{1st)}^{2}}$$

$$\text{Whiteness Index}\;=\;100\;‐\;\;\sqrt{{\left(100\;‐\;L\right)}^{2}\;+b^{2}\;+\;a^{2}}$$

$$\text{BI}\;=\;\frac{\left[\left(X\times 0.31\right)\times 100\right]}{0.172}\;,\;\text{where}\;\;X\;=\;\frac{a+1.75L}{5.645L+a‐0.3012b}$$

### 2.6 Starter and total bacterial count of UF‐Feta cheese

Two hundred gram of cheese was aseptically collected and homogenized in 180 ml of a 2% sodium citrate solution pH 8.75. Serial dilutions to 10^–8^ were prepared (in triplicate), and 100 µl were plating on selective media. The plate count agar (BD Difco^TM^) was incubated at 30°C and 72 hr for bacterial total count, and the M17 agar (BD Difco^TM^) was incubated at 25°C up to 5 days for mesophilic Lactococcus count (Vazquez‐Velazquez et al., 2018).

### 2.7 Sensory evaluation of UF‐Feta cheese after 60‐day ripening period

The sensory characteristics including, flavor, texture, after taste (salty, bitter and metallic taste), color, and overall acceptance (the average of the sensory evaluation parameters) of the UF‐Feta cheese samples were also determined using 20‐trained panelists. The panelists had a high level of discrimination, sensitivity, and consistency in measurement. The sensory evaluation was done on 60th day after cheese making as a seven‐point hedonic scale; the higher score means the higher quality (Afkhami et al., 2019; Nazari and Goli, 2017).

### 2.8 Statistical analysis

All tests in a completely randomized design based on two‐factor factorial using statistical SPSS software version 16 were conducted (Chicago, SPSS Inc.). The first factor was the type of salt in brine (i.e., sodium, potassium, calcium, and magnesium chloride), and the second factor was the ripening period after UF‐Feta cheese making (days 1 and 60). As a result, the present study performed with eight treatments and three replications. Duncan's multiple range test determined significant differences at the 95% confidential level (*p* < .05).

## 3 RESULTS AND DISCUSSION

### 3.1 Chemical analysis and bacterial count of UF‐Feta cheese

According to Table 1, acidity, pH, moisture, and total solid were significantly affected by either the type of brine or ripening period (*p* < .05). As the ripening period of the cheese increased, the acidity and total solid significantly increased while pH and moisture significantly decreased (*p* < .05). The pH value and acidity of UF‐Feta cheese samples were significantly affected by the ripening time (*p* < .05). During ripening period, the breakdown of proteins to nitrogen compounds by the proteolytic enzymes resulted in increasing the proteolysis index and pH and decreasing the acidity. As well as, the breakdown of lipids to free fatty acids by the lipolytic enzymes was been resulted in increasing lipolysis index, and the catabolism of lactose to lactic acid by the starter culture and total bacterial count that resulted in increasing the acidity and decreasing the pH (Dimitreli et al., 2017). The results of Table 1 showed that the type of salt consumed in the brine can significantly affect the chemical properties of UF‐Feta cheese (*p* < .05). The total substitution of NaCl with KCl, MgCl_2,_ and CaCl_2_ is associated with the production of cheese that is extremely sour and with alterations in texture, which resulted in the increase in the concentration of free fatty acids and proteolysis. The mixture 1:1 of NaCl and KCl, MgCl_2,_ and CaCl_2_ resulted in a cheese comparable to control (Cruz et al., 2011). Higher degree of lipolysis in Cheddar, Turkish White cheeses prepared with CaCl2, MgCl2, KCl, or blend of salts was observed (Khetra et al., 2019).With increasing the ripening period, the starter and total bacterial count showed a significant decrease and the highest inhibitory effect related to sodium and calcium salts (Table 1). All chloride salts exerted a significant antimicrobial effect on the LAB bacteria growth cycle; particularly, CaCl_2_ showed a similar effect to NaCl, while KCl and MgCl_2_ were progressively less inhibitory (Bautista‐Gallego et al., 2008). As the NaCl replacement with KCl increased the moisture, that is, a_w_, of Mozzarella cheese (Arboatti et al., 2014; Khetra et al., 2019), therefore, higher starter and total bacterial counts in potassium than sodium treatments can be justified. As well as, a higher number of the probiotic count, that is, *Lactobacillus casei*, in Iranian UF‐Feta cheese on substitution of NaCl with KCl, were counted (Karimi et al., 2012). In Cheddar, cheese fermentation of lactose to lactic acids was significantly influenced by salt‐to‐moisture ratio (Khetra et al., 2019; Upreti et al., 2006). As MgCl_2_ and CaCl_2_ cause the low salt‐to‐moisture ratio in UF‐Feta cheeses, therefore, higher starter and total bacterial counts in calcium and magnesium than sodium treatments can be justified.

**Table 1:** Comparison of the UF‐Feta cheese chemical properties, proteolysis and lipolysis index, starter and total bacterial count in different types of brine in days 1 and 60 after production

Day	Treatment	Acidity (%)	pH	Moisture (%)	Total solid (%)	Proteolysis index^a^ (%) (Water‐soluble *N* / Total *N*)	Lipolysis index^b^ (meq KOH/100 g cheese oil)	Starter bacterial count (log_10_ CFU/g)	Total bacterial count (log_10_ CFU/g)
1	NaCl	1.01 ± 0.03^e^	5.28 ± 0.02^a^	68.37 ± 0.01^c^	31.63 ± 0.01^d^	6.69 ± 0.03^e^	0.14 ± 0.03^e^	9.32 ± 0.01^b^	9.36 ± 0.01^b^
	KCl	1.05 ± 0.04^e^	5.26 ± 0.03^a^	68.47 ± 0.01^b^	31.53 ± 0.01^e^	6.65 ± 0.03^e^	0.15 ± 0.02^e^	9.42 ± 0.01^a^	9.47 ± 0.01^a^
	CaCl_2_	1.10 ± 0.01^d^	4.98 ± 0.04^b^	68.54 ± 0.02^a^	31.46 ± 0.02^f^	7.04 ± 0.03^e^	0.17 ± 0.04^e^	9.36 ± 0.01^b^	9.39 ± 0.01^b^
	MgCl_2_	1.10 ± 0.01^d^	5.02 ± 0.04^b^	68.35 ± 0.03^c^	31.65 ± 0.03^d^	7.05 ± 0.03^e^	0.17 ± 0.03^e^	9.45 ± 0.01^a^	9.45 ± 0.09^a^
60	NaCl	1.27 ± 0.01^c^	4.56 ± 0.01^c^	67.08 ± 0.01^f^	32.92 ± 0.01^a^	12.28 ± 0.01^b^	0.31 ± 0.01^d^	7.73 ± 0.01^f^	7.77 ± 0.08^f^
	KCl	1.30 ± 0.01^b^	4.55 ± 0.01^c^	67.35 ± 0.02^e^	32.65 ± 0.02^b^	11.08 ± 0.02^d^	0.41 ± 0.01^b^	7.88 ± 0.02^d^	7.90 ± 0.02^d^
	CaCl_2_	1.35 ± 0.01^a^	4.27 ± 0.01^e^	67.52 ± 0.03^d^	32.48 ± 0.03^c^	11.31 ± 0.01^c^	0.34 ± 0.01^c^	7.79 ± 0.01^e^	7.82 ± 0.09^e^
	MgCl_2_	1.31 ± 0.01^b^	4.43 ± 0.01^d^	67.04 ± 0.02^f^	32.96 ± 0.02^a^	12.41 ± 0.01^a^	0.47 ± 0.01^a^	7.93 ± 0.04^c^	7.95 ± 0.03^c^


Note

Mean ± *SD* values, followed by the same superscript letter within each column, are not significant differences in *p* ≤ .05 by ANOVA.

^a^ Or ripening index.

^b^ Or acid degree value (ADV).

### 3.2 Textural properties of UF‐Feta cheese

#### 3.2.1 Hardness and syneresis

The results of Table 2 clearly showed that after 60 days of cheese storage, the hardness and syneresis of the UF‐Feta cheese samples significantly decreased and increased, respectively (*p* < .05). The hardness of all samples reached its maximum on the first day and reached its minimum at the end of the 60‐day period. However, this trend was quite the opposite for syneresis. The results also showed that the effect of the type of salt on the hardness and syneresis parameters were significant (*p* < .05). The samples containing sodium and potassium chloride had a higher hardness and syneresis compared to the samples containing calcium and magnesium at every ripening period (Table 2). In addition, the highest hardness and syneresis on the first day seen in the samples containing sodium chloride and the lowest hardness and syneresis on the sixty day recognized in the samples containing magnesium chloride. In ion‐dependent structures, the placement of ions in the junction region increases the polymerization by creating an electrostatic equilibrium and thus increases the resistance of the probe to the penetration of the cheese structure (McMahon, 2010; McSweeney and OMahony 2016). In the early phase of the cheese storage period (first and second weeks after production), monovalent salts with a higher ability to reduce electrostatic repulsions create a charge balance between protein particles and facilitate interparticle interactions. Because of increasing interactions and polymerization of network chains, a structure with high‐molecular density and high strength was obtained. Therefore, we witnessed the highest degree of hardness on the first day of storage and in the presence of sodium and potassium salt, due to the formation of the most interaction between particles. On the other hand, regarding the comparison of the effect of monovalent salts, sodium salt has a lower ionic radius compared to potassium salt, which increases the polymerization and the appearance of higher hardness by affecting the reduction of space barrier between particles. In the second phase of the cheese storage period (from the second week onwards), the changes in the hardness of the samples are likely to be gradual with a decrease in density and the form of depolymerization due to increasing the intensity of proteolysis. On the other hand, increasing the replacement of sodium chloride with the other salts causes decreasing syneresis and consequently hardness. Therefore, by creating an electrostatic balance with the presence of monovalent salts and decreasing the level of divalent salts, we can see an increase in the level of interparticle interactions and create a harder texture in the cheese (Johnson & Lucey, 2006; McMahon, 2010).

**Table 2:** Comparison of the UF‐Feta cheese textural properties in different types of brine in days 1 and 60 after production

Day	Treatment	Syneresis (%)	Hardness (*N*)	Springiness	Cohesiveness	Adhesiveness (*N*. min)	Gumminess (*N*)	Chewiness (*N*)
1	NaCl	10.75 ± 0.02^e^	5.65 ± 0.05^a^	4.63 ± 0.05^b^	0.618 ± 0.001^a^	0.60 ± 0.01^a^	3.15 ± 0.01^a^	14.57 ± 0.16^a^
	KCl	10.1 ± 0.02^f^	5.10 ± 0.01^b^	4.34 ± 0.01^c^	0.521 ± 0.002^b^	0.59 ± 0.01^a^	2.94 ± 0.04^b^	12.76 ± 0.18^b^
	CaCl_2_	9.24 ± 0.02^g^	4.35 ± 0.05^c^	4.82 ± 0.02^a^	0.617 ± 0.022^a^	0.48 ± 0.01^c^	2.68 ± 0.12^c^	12.94 ± 0.65^b^
	MgCl_2_	8.80 ± 0.04^h^	4.00 ± 0.10^d^	4.72 ± 0.02^ab^	0.633 ± 0.016^a^	0.56 ± 0.01^b^	2.53 ± 0.01^d^	11.92 ± 0.03^c^
60	NaCl	17.85 ± 0.03^a^	3.40 ± 0.04^e^	4.04 ± 0.02^e^	0.470 ± 0.001^c^	0.27 ± 0.01^d^	1.60 ± 0.02^e^	6.44 ± 0.12^d^
	KCl	17.40 ± 0.05^b^	3.15 ± 0.05^f^	3.62 ± 0.02^f^	0.448 ± 0.009^d^	0.27 ± 0.02^d^	1.41 ± 0.01^f^	5.09 ± 0.04^e^
	CaCl_2_	16.84 ± 0.02^c^	2.25 ± 0.15^g^	4.11 ± 0.02^de^	0.478 ± 0.001^c^	0.14 ± 0.01^f^	1.07 ± 0.07^g^	4.41 ± 0.28^ef^
	MgCl_2_	16.22 ± 0.04^d^	1.90 ± 0.10^h^	4.23 ± 0.01^cd^	0.480 ± 0.002^c^	0.18 ± 0.01^e^	0.91 ± 0.05^h^	3.86 ± 0.23^f^

Note

Mean ± *SD* values, followed by the same superscript letter within each column, are not significant differences in *p* ≤ .05 by ANOVA. Gumminess applies only to semisolid products and is Hardness × Cohesiveness. Chewiness applies only to solid products and calculates as Gumminess × Springiness.

#### 3.2.2 Springiness

Changes in the springiness index, unlike the hardness index, were less strongly affected by the ripening period. The highest amount of springiness was observed in the first day of storage and in the level of magnesium chloride salt. Magnesium chloride as a divalent salt used as a cationic agent forms ion bridges between protein groups. The casein network of cheese was inherently formed by the binding of protein chains due to the presence of calcium ions. Therefore, the substitution of natural calcium chloride with magnesium chloride salt results in the substitution of magnesium ions for bonding in the casein network. The replacement of divalent magnesium ions in salt bridges within protein strands resulted in reducing the space barrier due to smaller radius of magnesium than calcium ions. The length of the protein network chain was shortened and increased flexibility, elasticity, and springiness index in samples of cheeses kept in brine containing magnesium chloride (Dabour et al., 2006; MacKintosh et al., 1995; Madureira et al., 2011).

#### 3.2.3 Cohesiveness

Cohesiveness was known as an indicator of the strength of the internal connections that form the texture of the product. This parameter also introduced as an indicator of product structural density. The results of Table 2 showed that with increasing ripening period, the cohesiveness of the UF‐Feta cheese decreased, which could be due to the increase in the proteolytic activity and proteolysis index (Table 1) (Gunasekaran & Mehmet, 2002). On the day 1 and 60 after production, the highest cohesiveness in the cheese sample containing sodium, calcium, and magnesium chloride was considered. In addition, using of potassium chloride weakened the cohesiveness of the cheese texture. Regarding the comparison of the effect of monovalent salts, sodium salt has a lower ionic radius compared to potassium salt, which increases the polymerization and the appearance of higher cohesiveness by affecting the reduction of space barrier between particles. The strength of the structural bonds of cheese is due to the formation of spherical aggregates between casein submicelles held together because of the formation of calcium or magnesium phosphate bonds with each other. Therefore, structural properties lead to the formation of calcium or magnesium phosphate bridges by affecting the density of microstructure, causing the cumulative mass of casein protein as the constituent tissue of cheese. In other words, the rearrangement of the casein mass structure is the result of aggregation between submicelles. The potassium chloride upset the relative equilibrium of the electrical charge between the protein particles by increasing the electrostatic repulsion between adjacent proteins and weakening the cohesiveness of the cheese texture (Griffin et al., 1988; Kakalis et al., 1990).

#### 3.2.4 Adhesiveness

The highest adhesiveness on the first day related to the sample containing sodium and potassium chloride and the lowest was for calcium chloride. Sodium and potassium chloride, due to their short ionic radius as well as their monovalent nature, cannot form crosslinks and help form an interconnected network in the texture. Therefore, reducing the level of crosslinks in the cheese casein network reduces cohesiveness and thus increases adhesiveness. The correlation coefficient between sodium, potassium, and calcium chloride salts with texture parameters of Halloumi cheese showed a positive relationship between the adhesiveness and sodium and potassium chloride salts and an inverse relationship with the presence of calcium chloride salt (Ayyash et al., 2011; 2012). Increasing the substitution level of monovalent salts with divalent salts caused promoting the formation of crosslinks between protein particles in cheese, which resulted in decreased adhesiveness (El‐Bakry et al., 2011). On the other hand, the adhesiveness of cheese texture significantly decreased with the increasing ripening period (Table 2). Based on the classification of the cheese ripening phase and the effect of that phase on the structure of the casein network and the rate of proteolysis during the long storage period, with the development of relative proteolysis, breakdown in protein chains and release of internal hydrophobic groups occurs. Accordingly, hydrophobic groups increase at the protein level and lead to the enhancement of hydrophobic interactions between particles, resulting in reduced adhesiveness of the sample during storage (Ayyash et al., 2011; El‐bakry et al., 2011).

#### 3.2.5 Chewiness

The chewiness parameter known as the energy index was used to break down the UF‐Feta cheese structure into smaller particles to prepare it for swallowing. Chewiness was applied only to solid products and calculated as gumminess multiplied by springiness (Zheng et al., 2016). The highest chewiness was observed in the sample containing sodium chloride salt on the first day and the lowest in the sample containing magnesium chloride salt after 60 days of storage (Table 2). The results of chewiness and hardness parameters were similar and both affected by the presence of sodium chloride salt in the structure. Based on the effect of sodium chloride salt on the hardness of UF‐Feta cheese texture and increase this parameter because of strengthening the structural density, therefore increasing the structural density due to the level of interactions between casein proteins in the structural network of cheese, caused to increasing the chewiness. Considering the relationship between chewiness and hardness, the chewiness of cheese has been more affected by the degree of texture hardness than the degree of its cohesiveness. Changes in the texture of Nabulsi cheese under the influence of sodium, potassium, calcium, and phosphate ions showed that the changes in chewiness and hardness were positively related to each other and affected by the amount of calcium and phosphate chloride in the structure. Moreover, by increasing the level of monovalent salts by removing the levels of calcium and phosphate salts in the cheese curd, the hardness and chewiness of the cheese texture increased (Ayyash & Shah, 2011).

### 3.3 Color assessment of UF‐Feta cheese

The results of Table 3 showed that during the 60‐day ripening period of UF‐Feta cheese in different brine, the highest color changes (i.e., ΔE) were related to salts other than sodium and no significant difference was observed between them. There was no significant difference in whiteness index for monovalent salts in the first and sixtieth days of storage, and of course, this difference was not significant between divalent salts. However, this index was statistically higher for divalent salts than for monovalent salts, especially sodium. This trend was the opposite of the whiteness index for the browning index. The highest browning index in the first and sixtieth days was related to divalent salts, and this index was not affected by the shelf life of cheese. The lowest browning index was found in cheeses kept in sodium and potassium brines, although this index was not affected by the ripening period of 60 days. Color is an important factor in consumer acceptance and directly related to product quality. It is influenced by how it reflects, absorbs, or transmits light, which in turn relates to the physical structure and chemical nature of the cheese (Kaya, 2002). The monovalent salts especially sodium chloride due to having a lower ionic radius and the reduction of space barrier between casein particles compared to calcium and magnesium chloride caused higher hardness and syneresis, which resulted in higher light reflection, that is, higher L* and whiteness index and lower browning index (McMahon, 2010; McSweeney and OMahony 2016).

**Table 3:** Comparison of the UF‐Feta cheese color assessment in different types of brine in days 1 and 60 after production

Day	Treatment	b*	a*	L*	ΔE(day 1st → day 60th)	Whiteness index	Browning index
1	NaCl	18.64 ± 1.03^a^	−6.64 ± 0.25^a^	95.69 ± 0.06^a^	–	79.73 ± 1.01^a^	15.66 ± 1.05^d^
	KCl	20.99 ± 3.42^bc^	−6.99 ± 1.20^bc^	92.55 ± 0.17^b^	–	76.63 ± 3.49^ab^	19.1 ± 3.57^cd^
	CaCl_2_	26.08 ± 6.06^ab^	−6.89 ± 2.58^ab^	88.70 ± 0.22^e^	–	70.61 ± 5.90^bc^	27.76 ± 6.75^ab^
	MgCl_2_	26.31 ± 0.44^ab^	−8.82 ± 0.31^ab^	91.87 ± 0.05^c^	–	71.08 ± 0.51^bc^	24.97 ± 0.38^abc^
60	NaCl	18.34 ± 0.75^a^	−7.06 ± 0.02^a^	96.21 ± 0.20^a^	1.12 ± 0.05^b^	79.96 ± 0.66^a^	14.84 ± 0.86^d^
	KCl	23.42 ± 1.19^abc^	−7.91 ± 1.57^abc^	92.42 ± 0.12^bc^	5.41 ± 2.54^a^	74.11 ± 1.52^abc^	21.54 ± 0.25^bcd^
	CaCl_2_	28.52 ± 1.82^a^	−6.88 ± 0.33^a^	86.64 ± 0.18^f^	5.45 ± 1.88^a^	67.75 ± 1.61^c^	32.14 ± 2.55^a^
	MgCl_2_	24.50 ± 1.53^abc^	−6.27 ± 0.52^abc^	89.42 ± 0.34^d^	4.05 ± 0.86^a^	72.57 ± 1.35^bc^	25.41 ± 1.65^abc^

Note

Mean ± *SD* values, followed by the same superscript letter within each column, are not significant differences in *p* ≤ .05 by ANOVA.

### 3.4 Sensory evaluation of UF‐Feta cheese

The panelists evaluated the sensory properties of UF‐Feta cheese samples (Table 4). The highest flavor score was related to calcium and magnesium brine treatments, although no significant difference was seen between these two treatments (*p* > .05) and there was a significant difference with the treatments containing sodium and potassium brine (*p* < .05). In addition, no significant difference was observed between the two treatments of sodium and potassium brine (*p* > .05). The diagnosis of panelists probably related to the trend of changes in acidity content, lipolysis index, and starter bacterial activity in treatments, which listed in Table 1. The highest texture score was related to sodium and potassium brine treatments, although no significant change was seen between these two treatments (*p* > .05) and there was a significant difference with the treatments containing calcium and magnesium salts (*p* < .05). As well as, no significant difference was observed between the two treatments of calcium and magnesium brine (*p* > .05). The diagnosis of panelists probably related to the trend of changes in hardness and solids content in brine treatments, which listed in Table 2. The previous studies showed that total substitution of sodium salt with potassium, calcium, and magnesium salt was associated with the production of cheese that is sour due to an increase in free acids concentration and with alterations in texture due to an increase in proteolysis index, which increased the flavor score and decrease in the texture score, respectively (Cruz et al., 2011). Salts modulate the flavor, texture, color, or appearance attributes of cheese by controlling the whey expulsion during manufacturing and growth of desirable and undesirable microorganisms and activity of enzymes during ripening (Khetra et al., 2019).

**Table 4:** The sensory evaluation of the UF‐Feta cheese samples on the day sixty after production (seven‐point hedonic scale, the higher score means the higher quality)

Treatment	Flavor	Texture	Taste			Color	Overall acceptability
			Salty	Bitter	Metallic		
NaCl	5.67 ± 0.37^cd^	6.20 ± 0.24^a^	6.32 ± 0.56^a^	6.45 ± 0.56^a^	6.35 ± 0.56^a^	6.40 ± 0.52^a^	6.32 ± 0.56^a^
KCl	5.80 ± 0.54^c^	6.05 ± 0.25^ab^	6.45 ± 0.24^a^	6.12 ± 0.24^ab^	6.15 ± 0.24^ab^	6.35 ± 0.45^a^	6.12 ± 0.24^ab^
CaCl_2_	6.15 ± 0.46^ab^	5.70 ± 0.35^cd^	4.85 ± 0.65^b^	5.55 ± 0.56^cd^	5.29 ± 0.56^cd^	5.95 ± 0.27^bc^	5.52 ± 0.56^c^
MgCl_2_	6.21 ± 0.42^a^	5.40 ± 0.37^d^	4.56 ± 0.36^b^	5.35 ± 0.56^d^	5.09 ± 0.45^d^	6.10 ± 0.14^b^	5.37 ± 0.56^cd^

Note

Mean values ± *SD*, (*n* = 20), Different letters in each column indicate significant differences (*p* < .05).

According to the taste sensory evaluation in terms of salty, bitter, and metallic taste attributes in which there was no significant difference between sodium and potassium brine treatments. As well as they had the highest taste sensory score, there was no significant difference between these two salts, compared to calcium and magnesium brine treatments, which had lower scores. There was no significant difference observed between the calcium and magnesium brine treatments (*p* > .05, Table 3). The substitution of NaCl by MgCl_2_ or CaCl_2_ caused off‐flavor (sour, bitter, metallic, and soapy) in cheeses except for KCl (Grummer et al., 2012; Khetra et al., 2019). The substitution of NaCl with high‐molecular weight compounds gives sour flavor and less saltiness in cheeses. Reduction or substitution of table salt causes uncontrolled enzymatic activity in reduced‐sodium cheeses, which results in the bitter flavor with soft body and texture (Khetra et al., 2019).

The data of color sensory evaluation in Table 4 showed that the highest color score was related to sodium and potassium brine treatments, although no significant change was seen between these two treatments (*p* > .05) and there was a significant difference with the treatments containing calcium and magnesium salts (*p* < .05). As well as, no significant difference was observed between the two treatments of calcium and magnesium brine (*p* > .05). The diagnosis of panelists probably related to the trend of changes in color assessment in brine treatments, which listed in Table 3. The monovalent salts due to having a lower ionic radius and the reduction of space barrier between casein particles compared to divalent salt caused higher hardness and syneresis, which resulted in higher light reflection, that is, higher L* and whiteness index and lower browning index (McMahon, 2010; McSweeney and OMahony 2016).

The overall acceptance score (the average of the sensory evaluation parameters) of the panelists showed that there was no significant difference between sodium and potassium brine treatments (*p* > .05), and these two treatments had a significant difference compared with calcium and potassium brine treatments (*p* < .05). There was no significant difference in the overall acceptance of cheeses stored in calcium and magnesium brine (*p* > .05). The sensory defects in the case of MgCl_2_ and CaCl_2_ were attributed to the low salt‐to‐moisture ratio in cheeses, which may cause uncontrolled enzymatic activity in the former that was not in the case of KCl. As well, in cheddar cheese making NaCl substitution with CaCl_2_ or MgCl_2_ caused an increase in bitterness with metallic flavor, whereas the texture was greasy with the crumbly and flaky body (Grummer et al., 2012; Khetra et al., 2019). In Halloumi cheese, acceptability characteristics did not differ significantly, though sensory scores decrease with an increase of KCl substitution (Kamleh et al., 2012; Khetra et al., 2019).

## 4 CONCLUSION

The antimicrobial effect of CaCl_2_ was the same as NaCl, while KCl and MgCl_2_ had progressively less inhibitory. The sodium chloride treatment caused the highest hardness and syneresis on the first day and the magnesium chloride on the sixty day caused the lowest hardness and syneresis. There was no significant difference in whiteness index for monovalent salts in the first and sixtieth days of storage, and of course, this difference was not significant between divalent salts. There was no significant difference in overall acceptance score between sodium and potassium brine, also between calcium and magnesium brine. Sodium chloride brine substitution with potassium, calcium, and magnesium chloride brine provides the dairy industry with the ability to cheese making with a variety of UF‐Feta cheeses to satisfying all tastes.

## 6 CONFLICT OF INTEREST

The authors declare that there are no conflicts of interest regarding the publication of this paper.

7

## Data Availability

The data that support the findings of this study are available from the corresponding author upon reasonable request.
